# Heat-tolerant hot pepper exhibits constant photosynthesis via increased transpiration rate, high proline content and fast recovery in heat stress condition

**DOI:** 10.1038/s41598-021-93697-5

**Published:** 2021-07-12

**Authors:** Sherzod Nigmatullayevich Rajametov, Eun Young Yang, Myeong Cheoul Cho, Soo Young Chae, Hyo Bong Jeong, Won Byoung Chae

**Affiliations:** 1 National Institute of Horticultural & Herbal Science, Rural Development Administration, Wanju, 55365 Republic of Korea; 2 Department of Environmental Horticulture, Dankook University, Cheonan, 31116 Republic of Korea

**Keywords:** Biochemistry, Physiology, Plant sciences, Environmental sciences

## Abstract

Understanding the mechanism for heat tolerance is important for the hot pepper breeding program to develop heat-tolerant cultivars in changing climate. This study was conducted to investigate physiological and biochemical parameters related to heat tolerance and to determine leaf heat damage levels critical for selecting heat-tolerant genotypes. Seedlings of two commercial cultivars, heat-tolerant ‘NW Bigarim’ (NB) and susceptible ‘Chyung Yang’ (CY), were grown in 42 °C for ten days. Photosynthesis, electrolyte conductivity, proline content were measured among seedlings during heat treatment. Photosynthetic rate was significantly reduced in ‘CY’ but not in ‘NB’ seedlings in 42 °C. Stomatal conductivity and transpiration rate was significantly higher in ‘NB’ than ‘CY’. Proline content was also significantly higher in ‘NB’. After heat treatment, leaf heat damages were determined as 0, 25, 50 and 75% and plants with different leaf heat damages were moved to a glasshouse (30–32/22–24 °C in day/night). The growth and developmental parameters were investigated until 70 days. ‘NB’ was significantly affected by leaf heat damages only in fruit yield while ‘CY’ was in fruit set, number and yield. ‘NB’ showed fast recovery after heat stress compared to ‘CY’. These results suggest that constant photosynthetic rate via increased transpiration rate as well as high proline content in heat stress condition confer faster recovery from heat damage of heat-tolerant cultivars in seedlings stages.

## Introduction

The heat stress caused by high temperatures induces morphological, anatomical, physiological, biochemical and genetic modifications in plants^1–4^. Photosynthetic and biochemical parameters can be good indicators for detecting effects of heat stress on plants because photosynthesis, growth and yield are closely related^5,6^. Therefore, it is vital to elucidate mechanisms of plants in response to heat stress, which may result in reducing reproductive success and destroying physiological activity, and consequently cause yield loss in agriculture^2–4^. Many plants have tolerance to heat stress but underlying mechanisms are not well understood yet^4,7,8^.

Hot pepper (*Capsicum annuum* L.) is one of the important vegetable crops of global significance. In changing climate, developing heat-tolerant cultivars has become a key research topic in the field of hot pepper breeding. In hot pepper, the temperature more than 30 °C not only significantly reduces the pollen viability, and fruit set^9,10^ and yield^11,12^, but induces the abscission of floral buds, flowers and immature fruits^11,13^. Various hot pepper accessions with diverse genetic backgrounds significantly differ in response to heat stress^14^.

A lot of screening methods for identifying heat-tolerant genotypes in different developmental stages and day and/or night temperatures have been developed for various crops in Solanaceae family^4,6,9,12,15^. Heat tolerance is a developmentally regulated, stage-specific phenomenon and its mechanism in hot pepper is still unknown. Heat tolerance at one developmental stage can be related^16^ or not to tolerance at the other developmental stages^17^. Therefore, breeding programs should focus on determining developmental stages susceptible to heat stress -to develop hot pepper cultivars with heat tolerance. Plants are usually susceptible to high temperature in early growth stages and, therefore, seedlings in the appropriate stage for transplanting can be proper starting materials to reveal heat tolerance mechanism in hot pepper.

The present study was conducted to understand the mechanisms to confer upon hot pepper plants the heat tolerance in seedling stages. Seedlings of two commercial pepper cultivars, one tolerant and the other susceptible to heat stress, were treated with severe temperature regime of 42 °C in day and night for ten days and their physiological changes were estimated. In addition, the growth and fruit yield parameters of seedlings with different leaf heat damages were investigated 70 days after heat treatment.

## Results

### Difference in physiological responses to heat treatment between heat-tolerant and susceptible seedlings

Plant height and shoot and root fresh weight were significantly reduced in seedlings grown in 42 °C compared to normal temperature regardless of heat-tolerant or susceptible cultivars (Supplementary Table S1). However, the heat-tolerant cultivar, ‘NB’ showed more reduction in shoot fresh weight than ‘CY’, the susceptible cultivar, but ‘CY’ showed more reduction in root fresh weight than ‘NB’ (Supplementary Table S1). Chlorophyll content significantly decreased as days of heat treatment (HT) increased in both cultivars (Supplementary Fig. S1) and the decrease was more prominent in a heat-susceptible cultivar, ‘CY’, than ‘NB’, after 2nd day of HT although the difference was not significant (see Supplementary Fig. S1).

**Figure 1:**
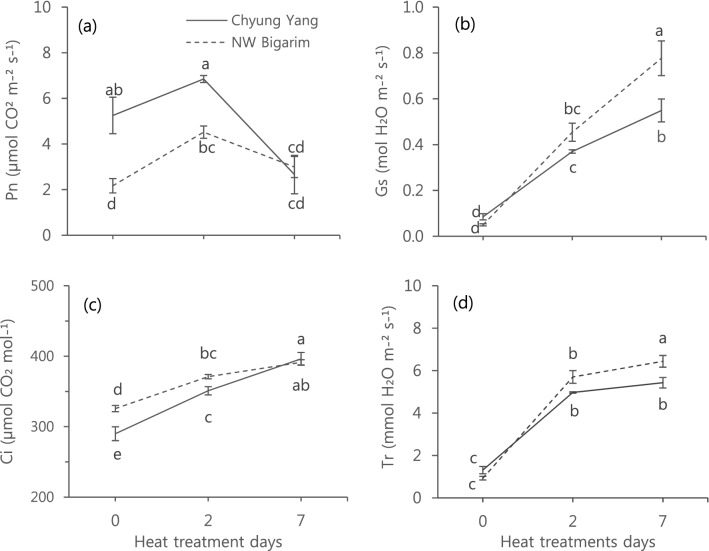
Effect of heat treatment of 42 °C on photosynthetic rate (**a**), stomatal conductivity (**b**), intercellular CO_2_ concentration (**c**) and transpiration rate (**d**) of hot pepper seedlings. Vertical bars represent standard deviation (n = 3). Different letters indicate significant differences by Duncan’s multiple range test at *p* < 0.05.

Photosynthetic rate was significantly reduced in a heat-susceptible cultivar compared to a tolerant one after seven days of HT (Fig. 1a). Although photosynthetic rate of ‘CY’ was significantly higher than that of ‘NB’ before HT, it decreased more in ‘CY’ than ‘NB’, after short increase in 2nd day, showing similar photosynthetic rate in both cultivars in 7th day of HT (Fig. 1a). Photosynthetic rate was not significantly different in ‘NB’, but significantly reduced in ‘CY’, between 0 and 7th days of HT (Fig. 1a).

The steady and significant increase in stomatal conductivity (Fig. 1b), the intercellular CO_2_ concentration (Fig. 1c) and transpiration rate (Fig. 1d) were observed in both cultivars when HT was prolonged. However, there were significant difference among them between two cultivars. Before HT, stomatal conductivity and transpiration rate was not different between two cultivars but significantly higher thereafter in ‘NB’ than ‘CY’, heat-tolerant and susceptible cultivars, respectively (Fig. 1b,d). In intercellular CO_2_ concentration, although there was initial significant difference between two cultivars before HT, the difference was not significant 7th day of HT (Fig. 1c).

**Figure 2:**
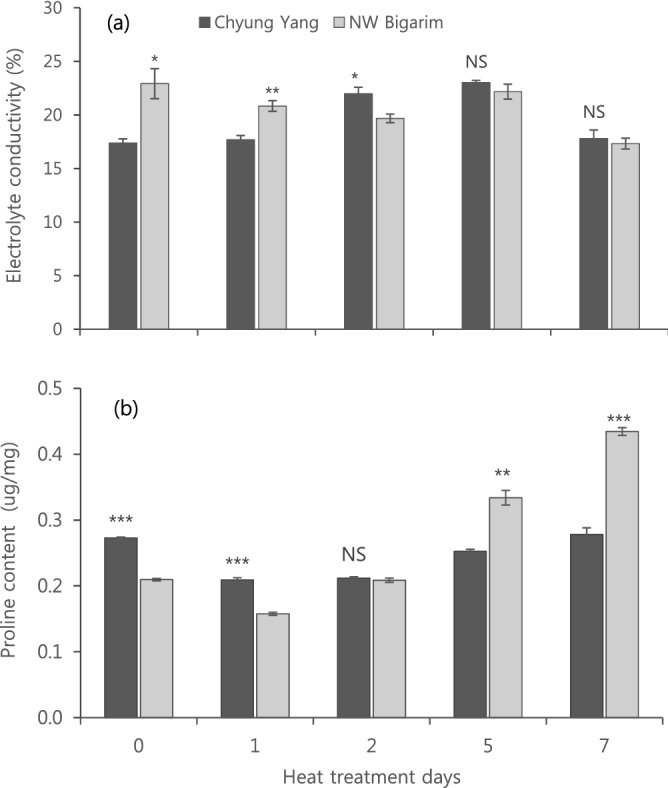
Electrolyte conductivity (**a**) and proline content (**b**) as affected by different heat treatment days in seedlings of two hot pepper cultivars grown in 42 °C. Vertical bars represent standard deviation (n = 3). NS, *, ** and *** indicate not significant and significant at the P < 0.05, P < 0.01 and P < 0.001 levels in t-test, respectively.

The cell membrane thermo-stability, calculated by electrolyte conductivity (EC), differ between two cultivars (Fig. 2a). EC was significantly higher in ‘NB’ before and 1st day of HT but reversed in 2nd day and no significant difference was observed between two cultivars after 5th day (Fig. 2a). However, there were different pattern in proline content (Fig. 2b). Proline content of ‘CY’ was significantly higher than that of ‘NB’ before and 1st day of HT but was significantly lower after 2nd day of HT than that of ‘NB’ (Fig. 2b). The proline content of ‘NB’ continuously increased from 1st to 7th days of HT but no distinct trend was observed in ‘CY’ (Fig. 2b).

### Effects of leaf heat damage levels on the growth and yield of heat-tolerant and susceptible cultivars

A leaf heat damage (LHD) increased significantly in both cultivars as days of HT increased (Supplementary Fig. S2). There was significantly difference in LHD in 10th day of HT, showing significantly higher in ‘NB’ (67.4%), a tolerant, than ‘CY’ (47.6%), a susceptible cultivar. It implies that ‘CY’ seems more tolerant to heat stress than ‘NB’ in seedling stages.

**Figure 3:**
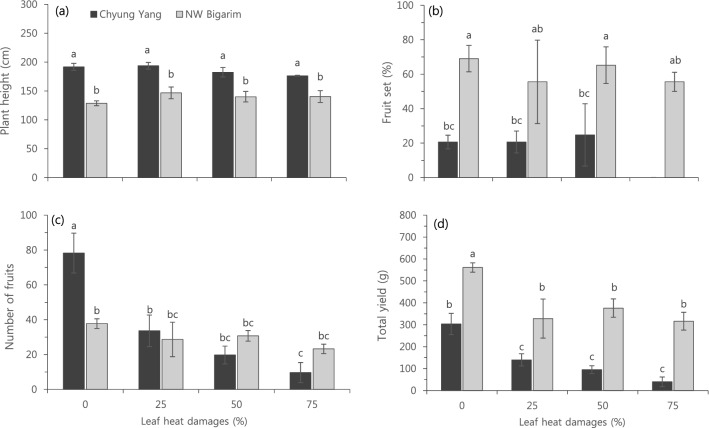
Effect of leaf heat damage levels on plant height (**a**), fruit set (**b**), the number of fruits (**c**) and total yield (**d**) in hot pepper cultivars. Vertical bars represent standard deviation (n = 3). Different letters indicate significant differences by Duncan’s multiple range test at *p* < 0.05. All parameters were measured on 70 days after transplanting.

The development, not the growth, of heat-tolerant and susceptible cultivars were significantly affected by LHD levels in the seedling stage (Fig. 3). Plant height in 70 days after transplanting (DAT) in a greenhouse (30–32/22–24 °C in day/night) was not affected by LHD levels although there was innate difference between two cultivars (Fig. 3a). In a heat susceptible cultivar, ‘CY’, LHD 75% significantly affected the fruit set while all LHD levels did not affect the fruit set of a heat-tolerant cultivar, ‘NB’ (Fig. 3b). The number of fruits in ‘CY’ was significantly affected by LHD levels, showing steady and significant decrease as LHD level increased (Fig. 3c). However, the number of fruits in a heat-tolerant cultivar, ‘NB” was not significantly affected by LHD levels (Fig. 3c). Total yield was significantly affected by LHD 25% in both cultivars but significantly higher in ‘NB’ than ‘CY’ in all LHD levels (Fig. 3d).

**Figure 4:**
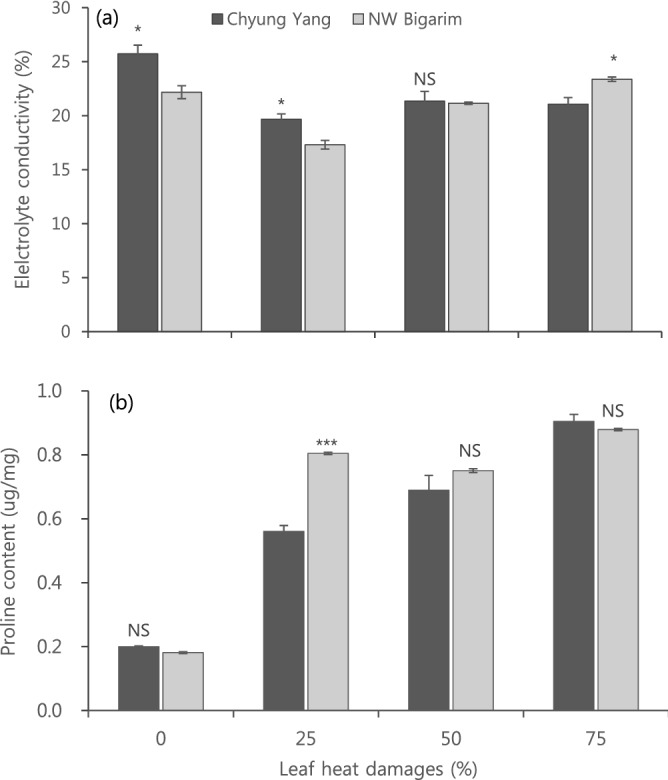
Electrolyte conductivity (**a**) and proline content (**b**) as affected by different leaf heat damages in seedlings of two hot pepper cultivars grown in 42 °C for 9 to 10 days. Vertical bars represent standard deviations (n = 3). NS, * and *** indicate not significant and significant at the P < 0.05 and P < 0.001 levels in t-test, respectively.

EC was significantly higher in ‘CY’ than ‘NB’ at LHD 0 and 25% but significantly lower at LHD 75% (Fig. 4a). There was no abrupt changes in EC in both cultivars, showing relative small changes within the range between 17 to 26% (Fig. 4a). However, the proline content increased as LHD levels increased from 0 to 75% (Fig. 4b). However, the differences between the two cultivars were not significant, except for LHD 25%, which was significantly higher in ‘NB’, a heat-tolerant cultivar.

**Figure 5:**
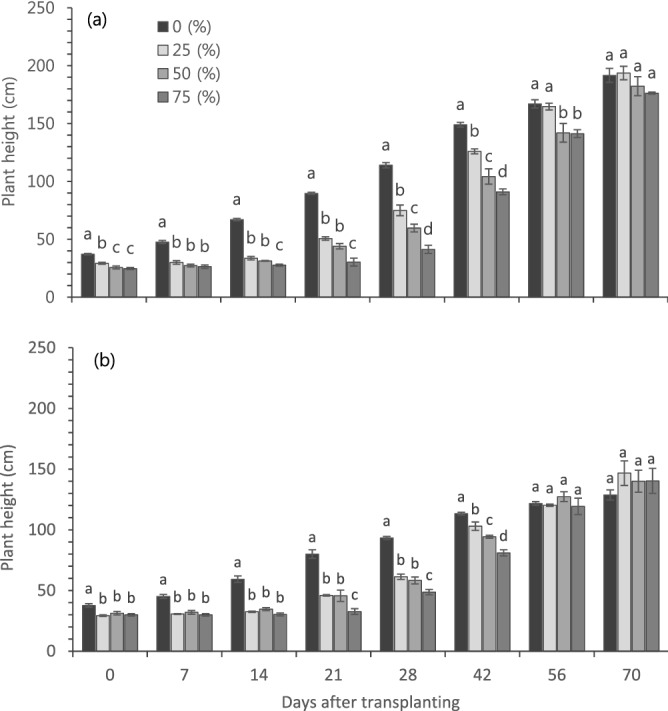
Effect of different leaf heat damage levels on plants growth rate in hot pepper cultivars susceptible (**a**, ‘Chyung Yang’) and tolerant (**b**, ‘NW Bigarim’) to heat stress. Vertical bars represent standard deviation (n = 3). Different letters indicate significant differences by Duncan’s multiple range test at *p* < 0.05.

**Figure 6:**
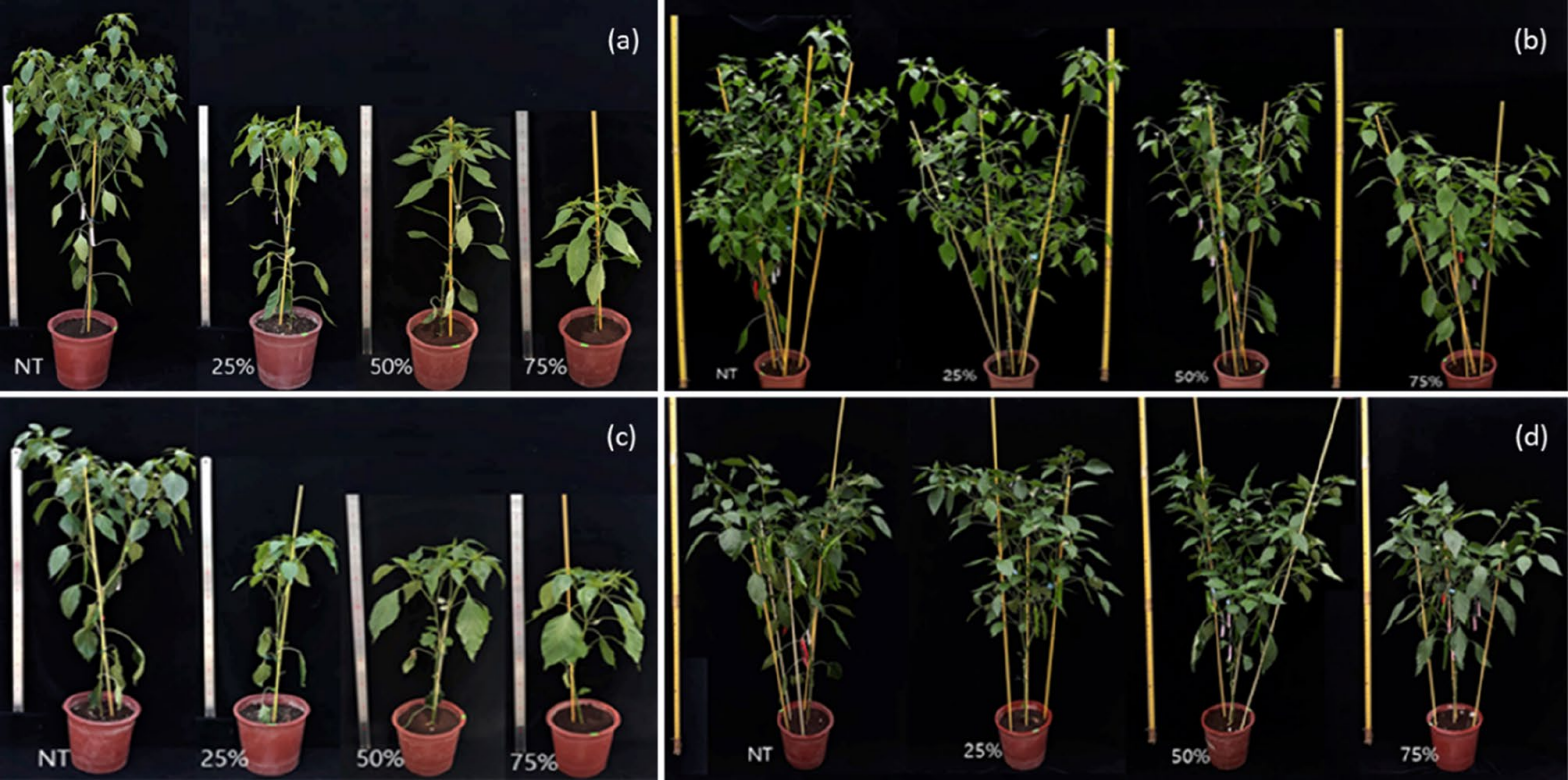
Effect of different leaf heat damages on the recovery of hot pepper plants. Cultivars ‘Chyung Yang’ in 30 days (**a**) and 55 days (**b**), and ‘NW Bigarim’ in 30 days (**c**) and 55 days (**d**) after heat treatment in 42 °C.

The plant growth was recovered faster in a heat tolerant than susceptible cultivar (Figs. 5, 6). The plant height was significantly lower in plants with LHD than those without LHD (plants did not go through HT) in ‘CY’ in early growth stages (Figs. 5a, 6a) and it was not until 70 DAT that all plants with different LHD levels showed similar plant height (Fig. 5a). ‘CY’ plants with LHD 25% were recovered earlier than other LHD levels (Fig. 5a). In contrast to ‘CY’, the plant height of ‘NB’ was not significantly different in 56 DAT among plants with LHD 0, 25, 50 and 70% (Figs. 5b, 6b), showing 14 days faster recovery than ‘CY’, except for LHD 25% (Fig. 5b).

## Discussion

Heat-tolerant cultivars are required to mitigate the adverse impact of changing climate without the yield reduction that was generally observed in heat susceptible cultivars^18^. The difference in heat tolerance can be related to different responses to high temperatures among genotypes and development stages^8,16,19^. The present study investigated physiological and biochemical changes due to high temperature using heat-tolerant and susceptible cultivars to understand the heat tolerance mechanisms in hot pepper seedlings.

The range and duration of high temperature influence the physiological parameters of plants^11,20,21^. Chlorophyll content in leaves decreased as HT days increased, and the decrease was faster in a heat-susceptible cultivar than a tolerant cultivar (Supplementary Fig. S1), as was observed in hot pepper^9,11^ and tomato^17,22^. The higher chlorophyll content in heat-tolerant cultivar gives better photosynthetic stability than heat-susceptible cultivars^16,17,22^.

In our study, HT significantly decrease photosynthetic rate in a heat-susceptible cultivar but not in a heat-tolerant cultivar (Fig. 1a). The stable photosynthetic rate of heat-tolerant cultivar in HT might be due to the increased stomatal conductivity (Fig. 1b) and transpiration rate (Fig. 1d). In tomato, high stomatal conductivity and transpiration rate under heat stress improves leaf cooling in heat-tolerant genotypes, providing better protection for chlorophyll and maintaining relatively high photosynthetic rate^16^. Also, a heat-tolerant cultivar had greener leaves than a heat-susceptible cultivar (see Supplementary Fig. S3) and this may contribute to better and stable photosynthesis^17^. The stay-green trait is also an important agronomical characteristic that contributes to higher yield under heat stress in tomato^23^.

Heat-tolerant cultivars may show more cell membrane thermostability than susceptible ones^2^ and cell membrane thermostability has been often linked to photosynthetic and transpiration rates^24^. However, membrane thermosstability seems not to contribute the photosynthesis and transpiration in hot pepper. Significant decrease in photosynthetic rate in a heat-susceptible cultivar (Fig. 1a) and significantly higher transpiration in a heat-tolerant cultivar (Fig. 1d) were observed while there was no difference in EC between both cultivars in 7th day (Fig. 2b).

In response to environmental stresses, plants accumulate osmoprotectants including proline^17,25^ and they are generally found in large amounts under any stress conditions^15,19,23^. Our results are in line with previous studies. Proline content increased as LHD increased regardless of cultivars (Fig. 4b). Interestingly, proline content that was significantly lower in early days of HT became higher in a heat-tolerant cultivar than heat-susceptible one from 5th day of HT (Fig. 2b). This indicate that heat tolerant cultivar responses more to the duration of HT than the magnitude of leaf heat damage in terms of proline accumulation.

HT significantly affect the plant height in early growth stages and it was recovered in later growth stages; however, the recovery was faster in a heat-tolerant cultivars than susceptible one (Fig. 5). This is possibly due to the stable photosynthetic rate in a heat-tolerant cultivar throughout the entire days of HT (Fig. 1a). The fruit set is the main indicator for screening the response of genotype on abiotic stress condition^1,23,24^, and heat stress also significantly affect fruit set in other Solanaceae crops including tomato^4,17,19,26,27^. In hot pepper, fruit set of the heat-tolerant cultivar was not affected by HT (Fig. 3b). In addition, the number of fruits was also not affected by HT in a heat-tolerant cultivar (Fig. 3c).

The heat-tolerant cultivar was not affected by LHD levels in terms of plant height (Fig. 3a), fruit set (Fig. 3b) and the number of fruits (Fig. 3c), total yield was significantly reduced in LHD 25% (Fig. 3d). Therefore, more than LHD 25% can be critical level for selecting heat-tolerant genotypes in seedling stages of hot pepper.

In conclusion, the present study suggests mechanism for heat tolerance in hot pepper via constant photosynthetic rate possibly due to increased stomatal conductivity and transpiration rate in high temperature, which can improve leaf cooling. The steady photosynthetic rate and high proline content in high temperature can provide faster recovery from heat damage in seedlings of heat-tolerant hot pepper plants, which resulted in stable fruit set and the number of fruits, followed by high yield. In addition, LHD levels over 25% in seedling stage was critical for selecting heat-tolerant genotypes in hot pepper breeding program.

## Materials and methods

### Plant materials and heat treatment conditions

The seeds of two commercial pepper cultivars ‘CY’ and ‘NB’, which are susceptible and tolerant to high temperature, respectively, were sown in 31st March, 2020 in plastic trays (52 × 26 cm in size, 6 × 6 cm cells with pot volume 5 L) containing 1:1 ratio of sand and commercial bed soil (Bio Sangto, Seoul, Korea) containing coco peat (47.2%), peat moss (35%), zeolite (7%), vermiculite (10.0%), dolomite (0.6%), humectant (0.006%) and fertilizers (0.194%). A liter of water was provided to each tray daily, and the trays were placed in a glasshouse (26/18 °C in day/night with relative humidity within 65–70%) in National institute of Horticultural and Herbal Science, Wanju, Korea.

Hot pepper seedlings with 8–10 true leaves were transferred on 11 May 2020 to growth chamber, maintaining day and night temperatures of 42 °C, light intensity 800 µmol m^−2^ s^−1^ for 16 h and 60–70% relative humidity. For each cultivar, a total of 32 seedlings were grown in the growth chamber for 10 days and watered twice a day (total two liters) to avoid drought stress. Plant height, shoot fresh weight and, root length and fresh weight were measured 10th day in HT with three replications.

### Photosynthesis in heat treated seedlings

The photosynthetic rate (μmol CO_2_ m^−2^ s^−1^), stomatal conductance (mol H_2_O m^−2^ s^−1^), intercellular CO_2_ concentration (μmol CO_2_ mol^−1^) and transpiration rate (mmol H_2_O m^−2^ s^−1^) were measured from 5 to 6th leaves of 0, 2 and 7 days after HT between 10:00–12:00 am. Data were recorded in three plants per cultivar using a portable photosynthesis measurement system (LI-6400, LI-COR Bioscience, Lincoln, NE, USA). Light response curves was set to 800 μmol m ^−2^ s^−1^, the leaf chamber temperature was set to 25 °C, and the intercellular CO_2_ concentration was maintained at 400 μmol mol^−1^. The photosynthetic rate was measured automatically at each irradiation level after 3–4 min light exposure^28^.

### Chlorophyll content, electrolyte conductivity, proline content in leaves of heat treated seedlings

All measurements for chlorophyll content, EC and proline content were conducted in 0, 1, 2, 5 and 7 days in HT with biological (three plants) and technical replications. Chlorophyll content were estimated using SPAD meter (Konica Minolta, Japan) in hot pepper leaves between 5–6 internodes with three technical replications.

EC in hot pepper leaves was measured according to Camejo et al.^2^ with minor modifications. Five leaves per seedling were perforated into discs with a radius of 5.5 mm, and each disc was placed in a 15-mL tube containing 10 mL of deionized water and then incubated on a shaking table at 25 °C for 30 min. At this time, the conductivity (EC_1_) of water was measured using a STARA-HB conductivity meter (Thermo Orion, Waltham, MA, USA). The tube was heated in a boiling water bath for 30 min and cooled at room temperature for 20 min, and then the conductivity (EC_2_) was measured again. Final EC content was expressed as the percentage of EC_1_/EC_2_.

Proline content in hot pepper leaves was measured using colorimetric assay^25^. Leaf samples from heat treated and non-treated seedlings were lyophilized (− 72 °C) in a freezer dryer (IlShin BioBase, Korea) for three days. Each leaf sample (100 mg in dry weight) was homogenized with 2 ml of 3% (w/v) aqueous sulfosalicylic acid solution. The homogenate was centrifuge at 14,000 rpm for 7 min and 1 ml of supernatant was transferred to 5 ml micro tubes containing 1 ml of acid ninhydrin. Acid ninhydrin was prepared by adding ninhydrin (2.5 g/100 ml) to solution containing glacial acetic acid, distilled water and 6 M ortho-phosphoric acid 85% at ratio of 6:3:1, receptively. The reaction mixtures were kept immediately in boiling water bath (95 °C) for one hour. The reaction was stopped (boiled micro tubes were kept) at 4 °C for 20 min and reading were taken at wavelength of 546 nm by spectrophotometer (EON, BioTek Instruments, USA).

### Evaluation of leaf heat damage levels among seedlings

LHD levels of hot pepper plants after 10 days of HT were identified according to the visual injuries. Leaf damage was calculated by measuring the percentage of leaf area that was dried or light yellow-white colored and classified into four levels of LHD 0% (no heat treatment), LHD 25% with leaf damages from 11 to25%, LHD 50% from 25 to 50% and LHD 75% from 50 to 75%. Seedlings with LHD 25% were collected in 9^th^ day and those with LHD 50 and 75% were collected in 10^th^ day of HT. After 9–10 days of HT, seedlings were transferred to glasshouse condition described above and maintained for three days to recover.

### Electrolyte conductivity, proline content, growth and yield of seedlings with different leaf heat damage levels

After three days of recovery, seedlings were transplanted to plastic pots with the same substrate described above and all plants were maintained in a glasshouse condition (30–32/22–24 °C in day/night). All plants were watered once a day and fertilized weekly with 1 L of water containing 1 mL of N-6, P-10 and K-5 (HYPONeXm, Japan). EC and proline content of seedlings with LHD 0, 25, 50 and 75% were measured eight days after HT with the same methods described above.

The measurement of plant height were started 70 days after transplanting (DAT) with seven days interval during the first four weeks and then every 14 days. Fruit set (FS, %) was calculated as follows:

$${\text{Fruit~set}}~\left( \% \right) = \frac{{{\text{The~number~of~fruits}}}}{{{\text{The~number~of~flowers}}}} \times 100$$

The number of fruits and flowers was determined by counting those from first to fourth internodes and total yield was determined by the sum of fresh weight of all fruits. All measurements were conducted with three biological replications.

### Statistical analysis

The arrangement of hot pepper plants was completely randomized. Analysis of variance were performed using the SAS Enterprise Guide 7.1 (SAS Institute Inc., NC, USA) for the data of the physiological and agronomical parameters. The mean values were compared with a significance level of 5% using Duncan’s multiple range test or Student’s *t*-test at the P < 0.05, P < 0.01 and P < 0.001 levels.

### Informed consent

This article does not contain any studies involving animals or human participants as objects of research.

## Supplementary Information

Supplementary Information.

## Data Availability

The datasets generated during and/or analyzed during the current study are available from the corresponding author on responsible request.

## Abbreviations

HT: Heat treatment
LHD: Leaf heat damage
EC: Electrolyte conductivity
DAT: Days after transplanting
