# Supplementary material for: Heat-tolerant hot pepper exhibits constant photosynthesis via increased transpiration rate, high proline content and fast recovery in heat stress condition
Source: Sci Rep. 2021 Jul 12;11:14328. doi: 10.1038/s41598-021-93697-5 (PMC8275607; doi:10.1038/s41598-021-93697-5)
Supplement: Supplementary file 1 — Supplementary Information. [file 41598_2021_93697_MOESM1_ESM.pdf]

Manuscript title: Heat-tolerant hot pepper exhibits constant photosynthesis via increased transpiration rate, high proline content and fast recovery in heat stress condition

Authors: Sherzod Nigmatullayevich Rajametov[1†], Eun Young Yang[1†], Myeong Cheoul Cho[1], Soo Young Chae[1], Hyo Bong Jeong[1], and Won Byoung Chae[2*]

Affiliation: [1]*National Institute of Horticultural & Herbal Science, Rural Development Administration, Wanju, 55365, Republic of Korea*

[2]*Department of Environmental Horticulture, Dankook University, Cheonan 31116, Republic of Korea*

Mailing addresses

Sherzod Rajametov, e-mail: sherzod_2004@list.ru

Eun Young Yang, e-mail: yangyang2@korea.kr

Myeong Cheoul Cho, e-mail: chomc@korea.kr

Soo Young Chae, e-mail: cotez@korea.kr

Hyo Bong Jeong, e-mail:bong9846@korea.kr

Won Byoung Chae, e-mail: wbcae75@dankook.ac.kr

[†] Equal contribution

***Correspondence**: Won Byoung Chae. Department of Environmental Horticulture, Dankook University, Cheonan 31116, Republic of Korea

Phone: 82-41-550-3642

Email: wbchae75@dankook.ac.kr

Supplementary Table S1. Effects of heat treatment on the growth of hot pepper seedlings with 8-10 true leaves grown in a growth chamber maintaining 42°C for 10 days. All the values are the mean ± standard deviations (n = 3).

Cultivar	Plant height (cm)		Diff. (%)	Root length (cm)		Diff. (%)	Shoot fresh weight (g)		Diff. (%)	Root fresh weight (g)		Diff. (%)
	NT	HT		NT	HT		NT	HT		NT	HT	
Chyung Yang	42.8 ± 2.1	30.2 ±0.9	-29.4	10.2 ± 0.4	12.0 ± 0.6	17.6	13.7 ± 0.7	9.7 ± 1.1	-29.5	2.7 ± 0.4	1.4 ± 0.2	-47.8
NW Bigarim	43.5 ±2.2	32.3 ± 2.2	-25.7	15.7 ± 0.3	15.0 ± 0.6	-4.3	16.1 ± 1.1	8.8 ± 0.9	-45.2	4.6 ± 0.3	3.7 ± 0.4	-19.6

NT- no treated plants, HT – heat treated plants.

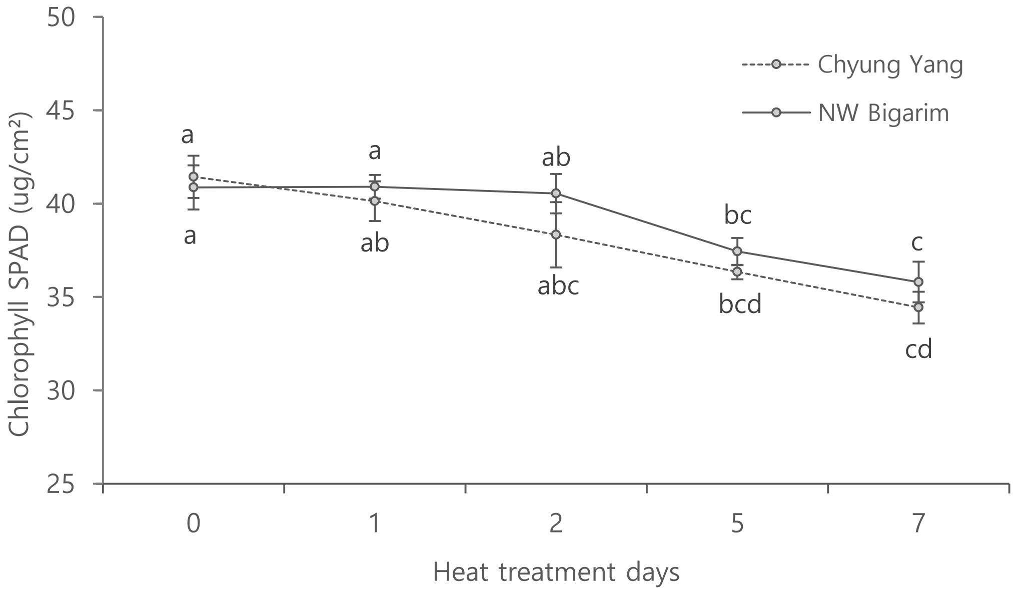

Supplementary Figure S1. Response of chlorophyll content in pepper leaves on heat stress. Vertical bars represent standard deviations (n = 3). Different letters indicate significant differences by Duncan's multiple range test at $p < 0.05$.

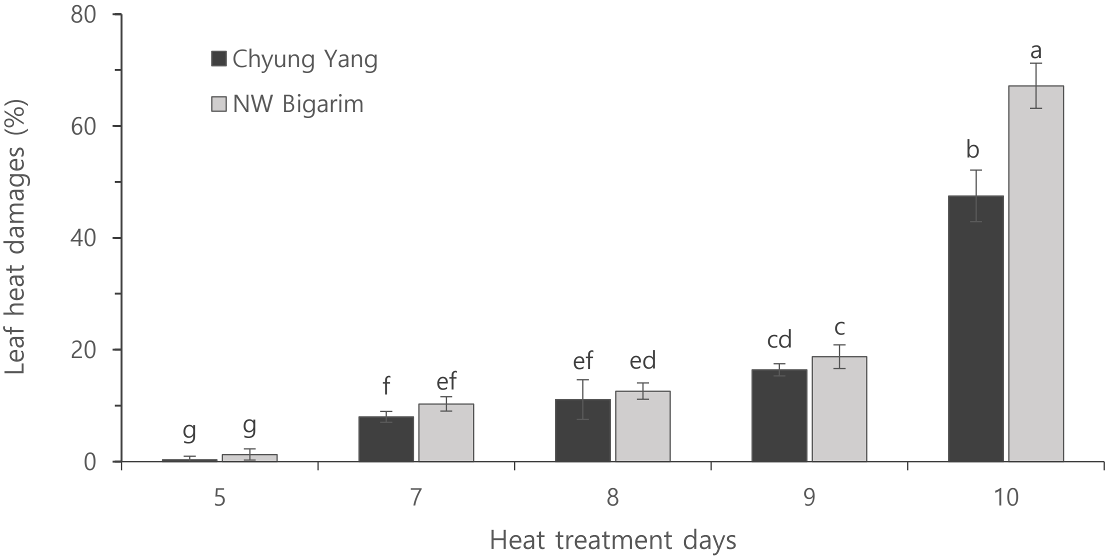

Supplementary Figure S2. Changes in leaf heat damages among pepper seedlings. Vertical bars represent standard deviations (n = 4). Different letters indicate significant differences by Duncan's multiple range test at $p < 0.05$.

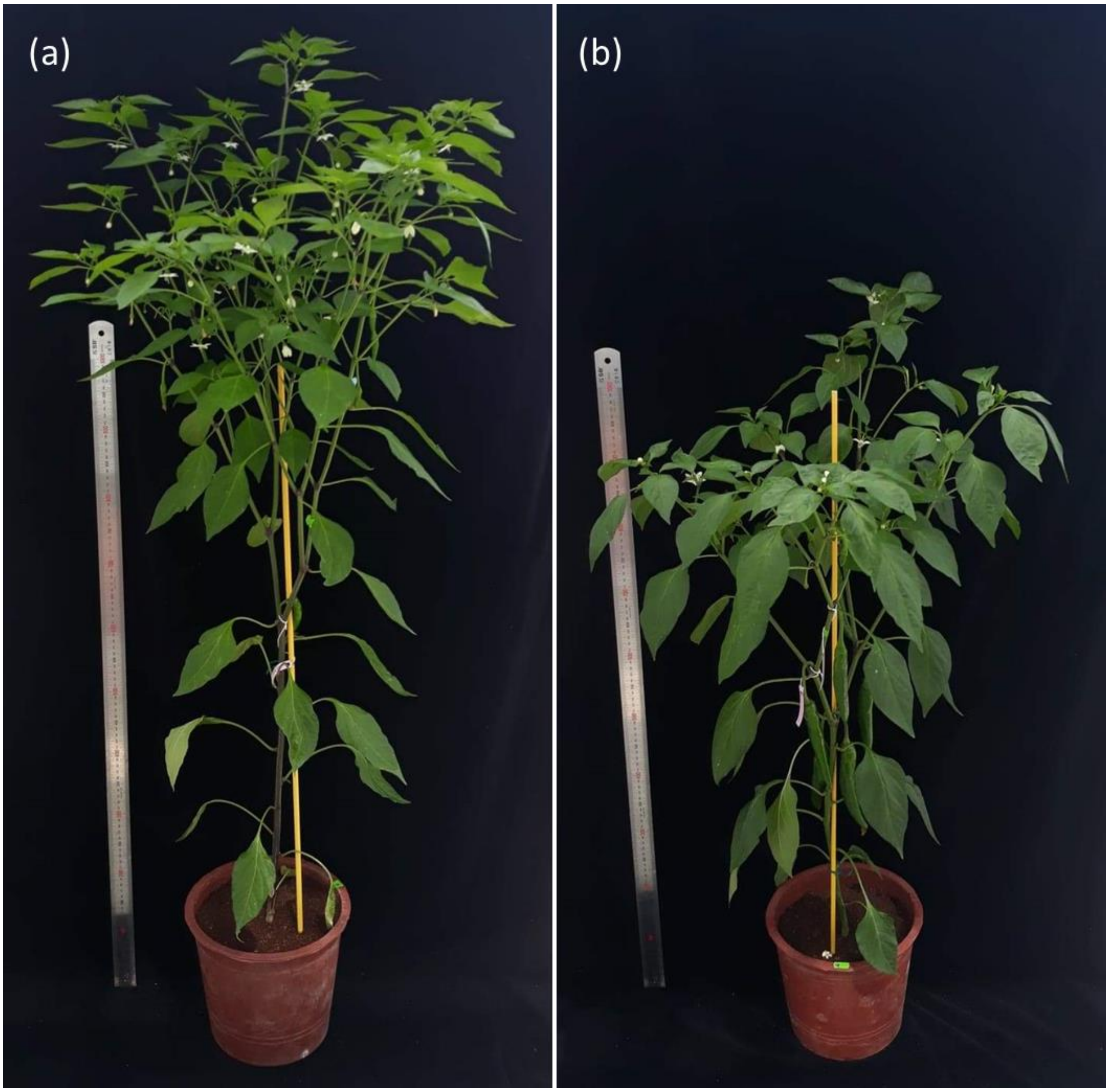

Supplementary Figure 3. Hot pepper plants 'Chyung Yang' (a) and 'NW Bigarim' (b) grown in normal temperature.
